# Rapidly Progressing Secondary Organizing Pneumonia Due to Underlying Immunosuppression With Rituximab in SARS-CoV-2 Patients

**DOI:** 10.7759/cureus.60328

**Published:** 2024-05-15

**Authors:** Madeline Vithya Barnaba Durairaj, Hayden Caraway, Robert Buresh, Rahul Sampath, Simon Waghchoure

**Affiliations:** 1 Internal Medicine, University of North Carolina (UNC) Health Blue Ridge, Morganton, USA; 2 Pathology, University of North Carolina (UNC) Health Blue Ridge, Morganton, USA; 3 Infectious Disease, University of North Carolina (UNC) Health Blue Ridge, Morganton, USA; 4 Intensive Care Unit, University of North Carolina (UNC) Health Blue Ridge, Morganton, USA

**Keywords:** lung, rituximab, covid-19, immunosuppression, secondary organizing pneumonia

## Abstract

Secondary organizing pneumonia (SOP) as a sequela to severe acute respiratory syndrome coronavirus 2 (SARS-CoV-2) usually has a prolonged benign course with a good response to corticosteroids. We present a case series of three patients who developed rapid progression to organizing pneumonia, after initial presentation with SARS-CoV-2. Imaging revealed rapid interval progression of bilateral subpleural ground glass opacities, and lung biopsy showed dense fibroblastic plugs within the alveoli. Two patients were steroid-responsive, and one patient succumbed to his illness despite maximal therapy. We postulate that B-cell depletion and immunosuppression may cause rapid progression to SOP, as all three patients were immunosuppressed and on chronic rituximab therapy.

## Introduction

The spectrum of clinical manifestations of pneumonia caused by severe acute respiratory syndrome coronavirus 2 (SARS-CoV-2) infection is diverse ranging from mild symptoms of cough, dyspnea, and fever to severe symptoms manifesting as respiratory failure secondary to underlying acute respiratory distress syndrome (ARDS) or organizing pneumonia [1]. Though diffuse alveolar damage (DAD) leading to ARDS has been the most common pathological finding in critically ill cases, patients with subacute presentations have secondary organizing pneumonia (SOP) or acute fibrinous and organizing pneumonia (AFOP) as the underlying pathology [2,3]. The risk factors for progression remain uncertain. We present a case series of three patients who developed SARS-CoV-2 pneumonia with rapid progression to SOP in the background of chronic rituximab treatment, an immunoglobulin monoclonal antibody that causes immunosuppression through B-cell depletion.

## Case presentation

Patient 1

A 56-year-old male presented to the emergency department (ED) with an acute onset of dyspnea progressing over two weeks. On examination, the patient was tachypneic at rest, saturating 85% on room air with bilateral basal crackles. On evaluation, he was found to be SARS-CoV-2-positive by polymerase chain reaction (PCR) test. Initially treated as an outpatient with bebtelovimab infusion without any improvement, he was later hospitalized for worsening dyspnea. Past medical history was significant for refractory polymyositis on immunosuppressive therapy with rituximab (diagnosed at 34 years of age), hypertension, and diabetes mellitus. He received his last dose of rituximab two weeks before presentation. A year ago, he had been vaccinated for SARS-CoV-2 with an mRNA vaccine consisting of two regular doses and a booster dose. Computed tomography (CT) of the chest showed bilateral lower lobe atelectasis with no classical signs of SARS-CoV-2 pneumonia. He was started on 3 L of oxygen via nasal cannula and a 10-day course of dexamethasone (6 mg once daily). He was also empirically treated for community-acquired pneumonia with azithromycin for three days (500 mg once daily) and ceftriaxone for five days (1 g every 24 hours). Remdesivir was not administered due to patient preference. He improved symptomatically and was discharged.

**Figure 1:**
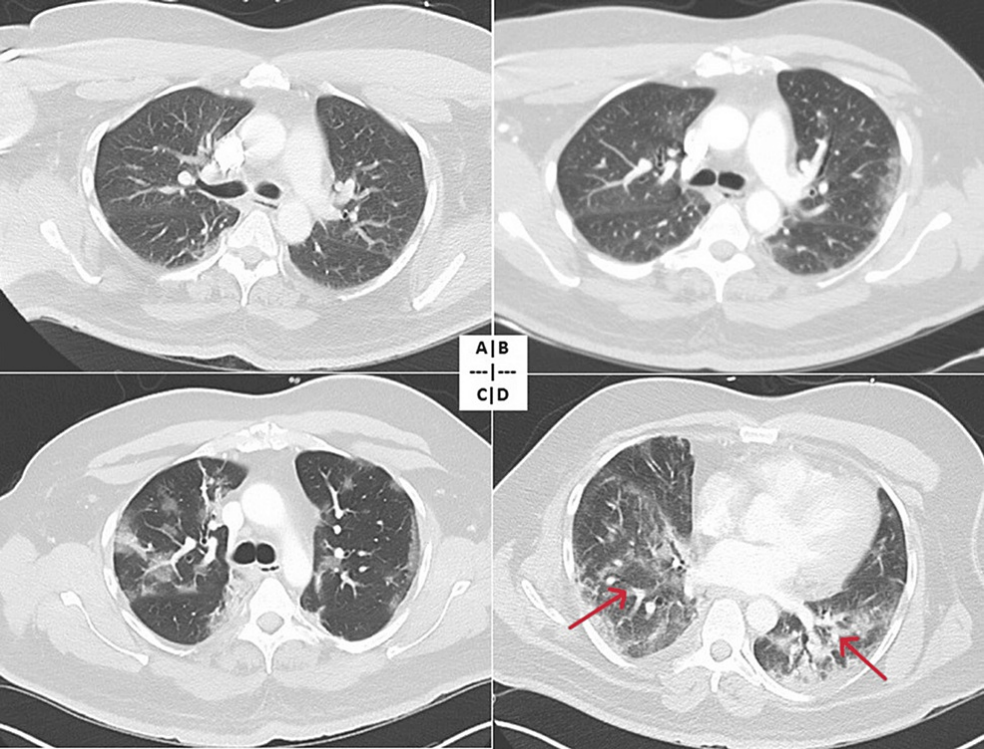
CT scan of the chest showing the development of secondary organizing pneumonia (patient 1). (A) CT of the chest on initial presentation with minimal ground glass opacities. (B) CT of the chest at two weeks showing interval development of bilateral ground glass opacities. (C) CT of the chest at three weeks with worsening bilateral ground glass opacities. (D) CT of the chest at four weeks with extensive bilateral consolidation (red arrows). CT: computed tomography

**Figure 2:**
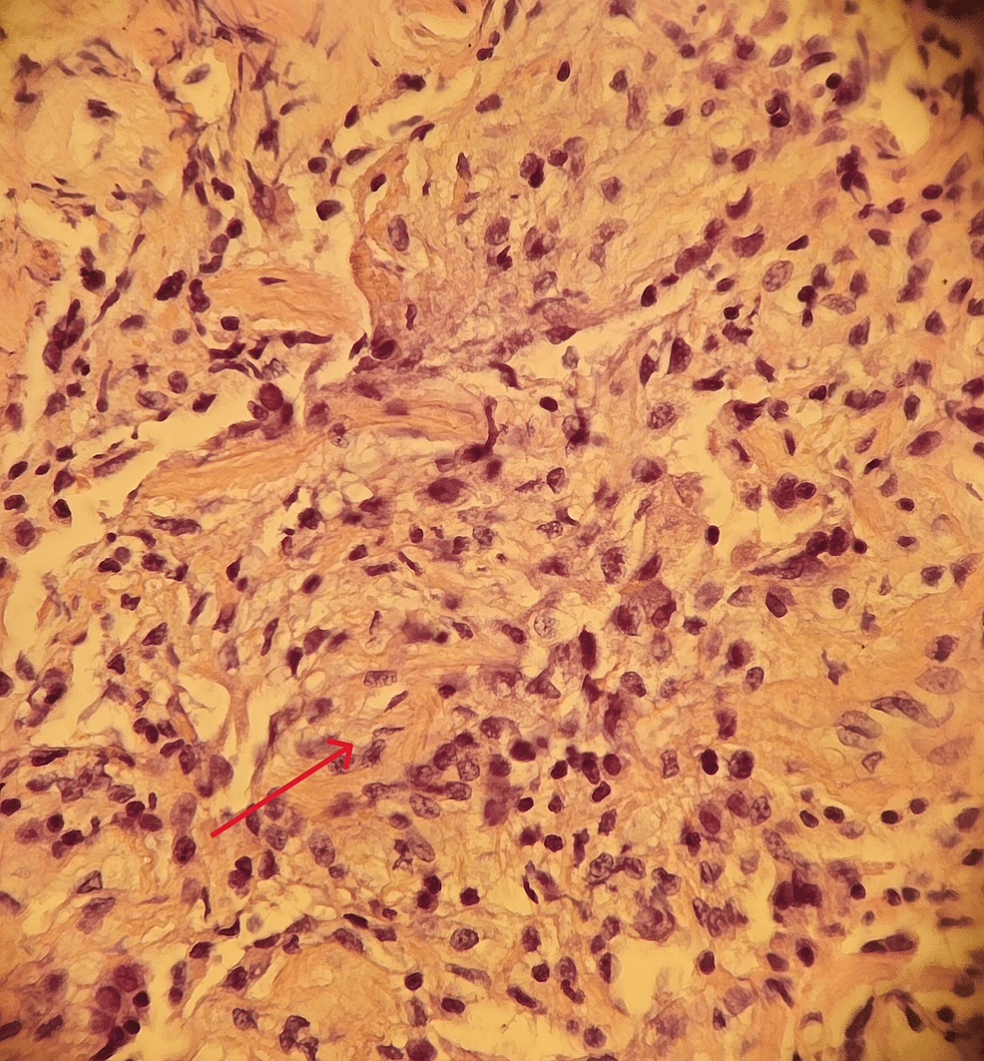
Dense fibroblastic plugs and spindle cells with an inflammatory background (red arrow), typical of organizing pneumonia.

Two weeks later, he was readmitted for high-grade persistent fevers for four days. On examination, he was found to be dyspneic with an oxygen saturation of 86% on room air. A respiratory examination revealed a bilateral wheeze. Infectious workup including respiratory pathogen panel, *Legionella* urine antigen, Fungitell, and respiratory cultures was negative. Repeat CT of the chest showed progressive peripheral subpleural ground glass opacities (Figure 1). Bronchoalveolar lavage showed mixed neutrophil-predominant inflammatory infiltrate with pulmonary macrophages. Transbronchial lung biopsy showed foamy macrophages and focal fibrosis suggestive of organizing pneumonia (Figure 2). Treatment was initiated with prednisone 1 mg/kg and then escalated to intravenous methylprednisolone. During hospitalization, he developed worsening crackles bilaterally and was put on a high-flow nasal cannula (HFNC) requiring a 100% fraction of inspired oxygen (FiO_2_) with an oxygen flow of 60 L per minute. Subsequently, he was put on mechanical ventilation and started on azathioprine. Despite maximal ventilatory support, there was minimal improvement. Hence, he was started on venovenous extracorporeal membrane oxygenation (ECMO) along with a lung rest strategy. Despite two weeks of continued treatment, he succumbed to his illness.

Patient 2

A 54-year-old male presented to the ED with a fever and persistent dry cough for five weeks. Four months back, he was found to be positive for SARS-CoV-2 (PCR) but remained asymptomatic. The symptoms started a month back when he was persistently SARS-CoV-2-positive. He was treated with dexamethasone and monoclonal antibody infusion (tocilizumab 8 mg/kg, single dose) along with antibiotics for suspected community-acquired pneumonia, which led to a slight interval improvement in symptoms, but they recurred. Past medical history was significant for mantle cell lymphoma (diagnosed four years back) on immunosuppressive therapy with rituximab, status post stem cell transplantation, in remission. He received his last dose of rituximab three weeks before presentation. He had been vaccinated for SARS-CoV-2 (non-mRNA vaccine) consisting of one regular dose, 18 months ago. He was a nonsmoker with no significant occupational exposure.

**Figure 3:**
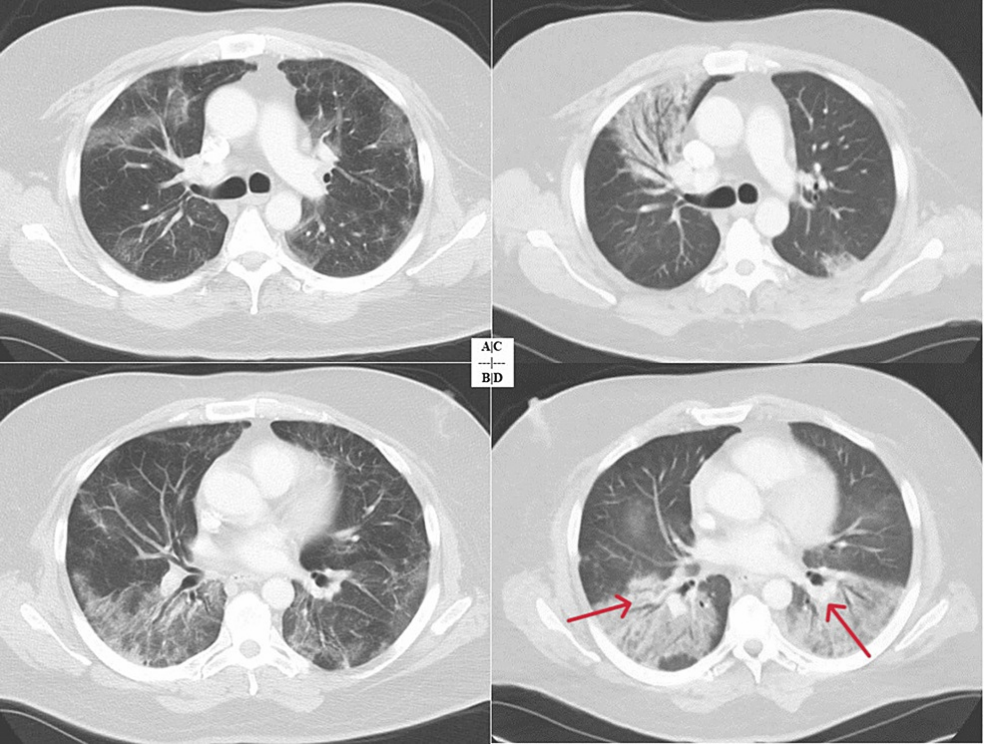
CT scan of the chest showing the development of secondary organizing pneumonia (patient 2). (A and B) CT of the chest showing bilateral subpleural ground glass opacities. (C and D) CT of the chest showing interval progression of bilateral ground glass opacities to consolidation (red arrows). CT: computed tomography

**Figure 4:**
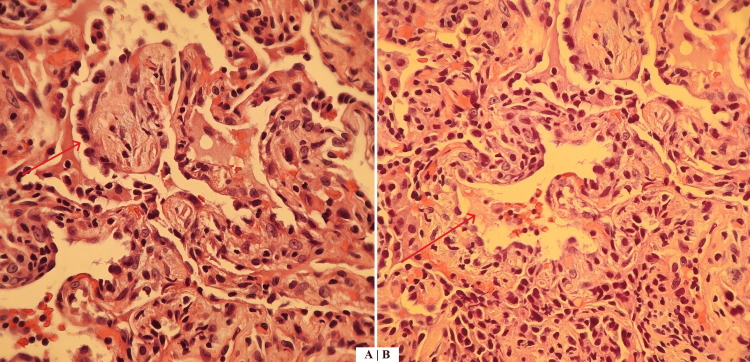
Transbronchial lung biopsy showing secondary organizing pneumonia (patient 2). (A) Cellular infiltration and fibroblastic plugs (red arrow) involving alveolar ducts are typically seen in organizing pneumonia. (B) Plugs of loose connective tissue protrude into the alveolar ducts and spaces (red arrow).

On admission, complete blood count (CBC) was unremarkable except for mild neutrophilic leukocytosis. Respiratory culture showed rare gram-positive cocci and gram-negative rods. Infectious workups including *Aspergillus*, *Legionella*, *Histoplasma*, *Blastomyces*, *Coccidioides*, *Pneumocystis*, *Cryptococcus*, HIV, and tuberculosis were negative. The autoimmune workup was negative. CT of the chest showed bilateral patchy ground glass opacities typically seen in SARS-CoV-2 pneumonia and a subsegmental right lower lobe pulmonary embolism (Figure 3). Bronchoalveolar lavage showed benign epithelial cells with macrophage-predominant mixed inflammatory infiltrate. The patient was treated with oxygen, dexamethasone, cefepime, and vancomycin for 10 days. Despite treatment, he developed progressive respiratory failure. Repeat CT of the chest showed the progression of infiltrates, and bronchoalveolar lavage sample testing was positive for SARS-CoV-2. Transbronchial lung biopsy showed fibroblastic plugs and the cellular infiltration of the alveoli suggestive of organizing pneumonia (Figure 4). He was continued on prednisone at 1 mg/kg with prolonged taper over 12 weeks. Over the next few months, he had two more hospitalizations requiring high-flow oxygen. At the six-month follow-up, he was doing well and had been gradually weaned off steroids and oxygen.

Patient 3

A 67-year-old female presented to the ED with an acute onset of dyspnea associated with weakness, nausea, vomiting, and diarrhea for one week. A month back, she was found to be SARS-CoV-2-positive (PCR) and had been treated with dexamethasone (6 mg for 10 days) along with nirmatrelvir and ritonavir. Past medical history was significant for rheumatoid arthritis (diagnosed around 20 years back) on immunosuppressive therapy with rituximab and mucosa-associated lymphoid tissue (MALT) lymphoma in remission. She received her last dose of rituximab three months before presentation. She was a chronic smoker with a 60-pack-year history and had received a SARS-CoV-2 (mRNA) vaccination consisting of two regular doses and a booster dose, two years ago.

**Figure 5:**
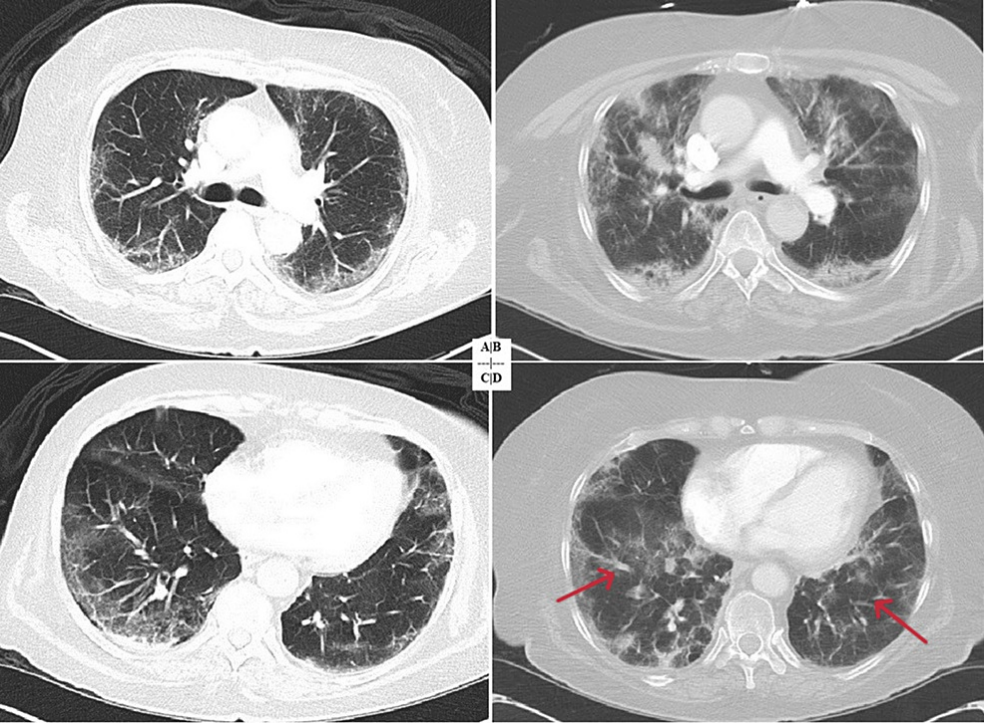
CT scan of the chest showing the development of secondary organizing pneumonia (patient 3). (A and C) CT of the chest showing bilateral subpleural ground glass opacities. (B and D) CT of the chest showing interval progression of bilateral ground glass opacities to consolidation (red arrows). CT: computed tomography

On admission, she was hypoxic, requiring 3 L of oxygen via nasal cannula. Examination revealed occasional bilateral crepitations. Investigations including CBC, comprehensive metabolic panel (CMP), respiratory pathogen panel, and respiratory culture were unremarkable. The infectious workup for typical and atypical respiratory pathogens was negative. The autoimmune workup was negative. CT of the chest showed peripheral subpleural ground glass opacities bilaterally. She was started on prednisone at 1 mg/kg for suspected secondary organizing pneumonia. Bronchoscopy was not done due to patient preference. She improved clinically with the resolution of lung signs and was discharged with 2 L of oxygen. Two days later, she was readmitted for worsening respiratory distress and needing high-flow oxygen. Sputum culture grew *Enterococcus*. Repeat CT of the chest showed interval progression of bilateral lung infiltrates and worsening consolidation (Figure 5). She was maintained on prednisone along with the addition of antibiotics. She gradually improved and was discharged to a pulmonary rehabilitation facility with 3 L of nasal oxygen. Two weeks later, she had a repeat hospitalization for methicillin-resistant *Staphylococcus aureus* (MRSA) pneumonia due to worsening dyspnea, which resolved with treatment (vancomycin). At the three-month follow-up, prednisone had been tapered and stopped along with the weaning of nasal oxygen.

## Discussion

Organizing pneumonia is pathologically characterized by intra-alveolar plugs of granulation tissue composed of fibroblasts and myofibroblasts intermixed with loose connective tissue [1]. Though often found to be idiopathic, commonly known as cryptogenic organizing pneumonia (COP), various etiologies have been shown to contribute to the development of secondary organizing pneumonia. Drugs, toxins, infections, radiation, and aspiration constitute some of the well-known etiologies [4,5]. Viral infections including adenovirus, influenza virus, parainfluenza virus, herpes, and HIV have been commonly implicated. However, very few case studies have reported the association between SARS-CoV-2 infection and the development of secondary organizing pneumonia (SOP). Studies have shown that SOP usually has a benign course with slow progression and a good response to corticosteroids [6]. We present a case series of three immunocompromised patients with a recent history of SARS-CoV-2 infection who had rapid progression to secondary organizing pneumonia.

Initial direct antigenic insult to the alveoli and endothelial cell layers results in an early acute exudative phase characterized by the accumulation of fluid and protein within the alveoli leading to diffuse alveolar damage (DAD), which has been observed in a majority of patients [7]. In a minority of cases, alveolar epithelial injury ensues leading to a proliferative/organizing phase characterized by the leakage of fibrin [2,8]. Acute fibrinous and organizing pneumonia (AFOP) is closely related to organizing pneumonia, with an intra-alveolar accumulation of fibrin being the dominant pathological finding [2,3,9]. The organizing and fibrotic stages of pneumonia are more often seen in prolonged cases of SARS-CoV-2 probably secondary to persistent insult due to underlying smoldering infection despite initial treatment.

Epidemiological studies have shown that the severity of the presentation of SARS-CoV-2 pneumonia correlates with age and underlying comorbidities [10]. Typically, secondary organizing pneumonia secondary to SARS-CoV-2 has a subacute presentation, although rapidly progressive fulminant respiratory failure has been described in 5%-8% of cases [5]. Recent history of smoldering SARS-CoV-2 infection in the setting of immune-modulating medications may have led to a rapidly progressive clinical course with worsening respiratory failure in our patients with organizing pneumonia. Radiological consolidation in the subpleural or peri-broncho vascular distribution has been linked to organizing pneumonia, although a mixed pattern of ground glass opacities along with consolidation is possible as well [11]. The typical radiological appearance that manifests in SARS-CoV-2 patients is bilateral subpleural ground glass opacities with air bronchograms and ill-defined margins [12]. SOP is usually associated with diffuse bibasilar consolidation and ground glass opacities such as DAD [13].

We hypothesize that the relatively rapid progression of underlying organizing pneumonia due to persistent viremic insult from SARS-CoV-2 leads to the transition of acute lung injury into organizing pneumonia in our patients. This may have been precipitated by chronic immunosuppression with rituximab, an immunoglobulin monoclonal antibody against cluster of differentiation 20 (CD20) causing B-cell depletion [14]. We postulate that chronic immunosuppression with rituximab may have led to increased viremia, increased lung injury, and the rapid progression of the organizing pneumonia in these vaccinated patients.

Corticosteroids, mostly prednisone, are considered the standard treatment for secondary organizing pneumonia [1-3]. There is usually marked improvement in symptoms in response to treatment, although relapses are quite common. The lack of response may indicate progression to the fibrotic stage with the development of organizing pneumonia. Treatment for organizing pneumonia secondary to SARS-CoV-2 seems to be similar; however, higher dosage and longer duration of treatment may be necessary [15,16]. Adjunct treatment with immunomodulators such as azathioprine and cyclophosphamide has been tried, though the evidence is scarce [3]. One of our patients did receive azathioprine due to inadequate response to steroids, albeit with no clinical improvement. Furthermore, few case reports exist that have shown significant clinical improvement with a prolonged course of antiviral treatment [17,18].

## Conclusions

In conclusion, a growing body of evidence suggests that secondary organizing pneumonia needs to be strongly considered in the differential of patients with SARS-CoV-2 pneumonia presenting with persistent or relapsing respiratory symptoms and progression of radiological features. Whether underlying immunosuppression remains a risk factor for rapid progression to organizing pneumonia is unknown. Further research is mandated to ascertain a causal association with these risk factors. Corticosteroids remain the cornerstone of therapy, although more research is needed to determine if long-term antiviral treatment against SARS-CoV-2 may be of benefit to this population.
